# A Friend in Prison: Human-Animal Bond, Stress and Self-Esteem of Detained Juveniles in Dutch Cell Dogs

**DOI:** 10.3390/ani12050646

**Published:** 2022-03-03

**Authors:** Esther M. Karkdijk, Hanne M. Duindam, Maja Deković, Hanneke E. Creemers, Jessica J. Asscher

**Affiliations:** 1 Research Institute of Child Development and Education, University of Amsterdam, 1018 WS Amsterdam, The Netherlands; h.e.creemers@uva.nl (H.E.C.); j.j.asscher@uu.nl (J.J.A.); 2 Clinical Child & Family Studies, Utrecht University, 3584 CS Utrecht, The Netherlands; h.m.duindam@uu.nl (H.M.D.); m.dekovic@uu.nl (M.D.)

**Keywords:** prison-based dog training program, human–animal bond, detained juveniles, stress, self-esteem

## Abstract

**Simple Summary:**

Interventions that aim to increase well-being among detained juveniles, in addition to interventions focusing on behavioral change, are urgently needed and should be increasingly implemented. A promising and popular intervention is the prison-based dog training program. In such a program, detainees train shelter dogs to prepare them for adoption. In literature about these interventions, it is often assumed that the perceived bond with the dog plays an important role in improving well-being among detainees. For example, it is assumed to decrease stress and increase self-esteem. However, the human–animal bond within a prison-based dog training program and its effects are seldom investigated. In this study, we investigated to what extent the human–animal bond predicts stress and self-esteem among detained juveniles, participating in a prison-based dog training program in the Netherlands (Dutch Cell Dogs). Questionnaires and interviews at several timepoints were used to measure the quality of the human–animal bond, and the perceived reciprocity, stress, and self-esteem. The results of this study show that the human–animal bond did not predict lower stress or higher self-esteem, contrary to our expectations. More research on prison-based dog training programs is needed to investigate how these programs work, and the specific role of the human–animal bond within these programs.

**Abstract:**

This study examined to what extent the human–animal bond (HAB) had a positive impact on stress and self-esteem among detained juveniles participating in the prison-based dog training program Dutch Cell Dogs (DCD). Participants were 75 detained juveniles (mean age = 19.5, 86.7% male). Self-reported stress and self-esteem were assessed before the start of DCD (T1), after four weeks (halfway training/T2) and after eight weeks (end training/T3). Structured interviews and questionnaire items were used to measure the HAB quality and perceived reciprocity in the HAB at T2 and T3. Data were analyzed using Structural Equation Modeling. In the variable-centered approach analyses, only the cross-sectional positive association between HAB quality and self-esteem at T2 was significant in the cross-lagged panel models. None of the cross-lagged paths between the HAB and stress or self-esteem were significant. In the person-centered approach analyses, growth mixture modeling identified two patterns of self-esteem (“high stable” and “high decreasing”); however, these patterns were not predicted by HAB. Thus, in contrast to our hypotheses, the HAB did not predict improvements in detained juveniles’ stress and self-esteem. These findings underline the need for more research into the often-presumed role of HAB within prison-based dog training programs.

## 1. Introduction

Detained juveniles represent a high-risk, vulnerable population [1]. Being detained has been associated with several adverse outcomes, such as loneliness and depression [2,3]. The prevalence of mental health disorders among detained juveniles is alarmingly high: 60 to 70 percent meets the diagnostic criteria for at least one mental health disorder [4,5]. Therefore, interventions that aim to increase well-being among detained juveniles, in addition to already existing interventions focusing on behavioral change, are urgently needed and increasingly implemented [6,7].

### 1.1. Prison-Based Dog Training Programs

One promising intervention to improve well-being of detained juveniles is the Prison Based Dog Program (PBDP). In PBDPs, dogs are included to improve wellbeing and stimulate positive behavioral change in detainees [8]. In the community-service Dog-Training Program (DTP), detainees train behaviorally challenging shelter dogs to prepare them for adoption [9]. These programs are unique in providing detainees with opportunities to be responsible and caring [9]. In contrast to many other prison-based interventions, the main focus of these programs is on helping the dog, instead of on the detainees [10].

Dutch Cell Dogs (DCD) is a DTP in the Netherlands in which (juvenile) detainees train shelter dogs to equip them for rehoming. The dogs are brought to the correctional facilities twice a week for two-hour training sessions, for eight succeeding weeks. An important aim is to socialize the dogs and thereby improve their adoption chances. Although there are no explicit therapeutic goals for the detainees, the program intends to improve their wellbeing. Furthermore, it is expected that by training the behaviorally challenging dogs, detainees develop social and communication skills and their self-esteem increases [11]. Therefore, DCD aims to create a win–win situation for the shelter dogs and detainees.

DTPs, such as DCD, can differ from other PBDPs with respect to the program’s focus and intensity. For example, in other PBDPs, dogs are mainly included to facilitate the achievement of detainees’ therapeutic goals [9] or are more integrated in detainees’ life (e.g., residing with participants).

### 1.2. Effectiveness of DTPs

Positive outcomes of DTPs have been described. Cooke and Farrington [12] concluded in a meta-analysis that DTPs had a significant, but relatively small effect on externalizing and internalizing outcomes, such as self-esteem. However, the findings should be interpreted with caution, given that many studies had methodological limitations (e.g., small samples or no control group). A recent meta-analysis, including only studies with a quasi-experimental or randomized controlled trial design, showed a small overall effect of PBDPs [13]. This finding, which was irrespective of program type, was largely driven by effectiveness in reducing criminal recidivism. However, no significant effect for social-emotional functioning was found.

Recently, a quasi-experimental study examined the short-term effectiveness of DCD in juveniles [14]. In line with the pilot study with a multiple case design [15], nonsignificant effects on aggression, institutional infractions, wellbeing, and therapeutic alliance and motivation were found. However, the findings show heterogeneity in response to DCD, related to facility type and cultural background. The authors stressed that more research into the working mechanisms and moderators of effectiveness is needed to determine how, and for whom, DTPs may work.

Although various studies regarding the effectiveness of PBDPs have been carried out, it is remarkable that research into the underlying mechanisms is largely lacking. The present study aimed to expand knowledge of DTPs’ underlying mechanisms, by examining the role of the human–animal bond (HAB). Data of the study of Duindam and colleagues [14] were used to examine the association between HAB, on one hand, and stress and self-esteem on the other hand, among detained juveniles participating in DCD.

### 1.3. The Human–Animal Bond and Psychosocial Well-Being

One frequently proposed mechanism in DTPs is the HAB: the social attachment between people and their companion animals [16]. Various studies show physiological and psychological benefits of human–animal interaction [17]. In this study, we focused on two frequently reported outcomes of PBDPs: decrease in stress, e.g., [18] and increase in self-esteem, e.g., [12].

#### 1.3.1. Stress

By offering non-judgmental and unconditional positive regard, dogs may provide social support [19]. The experience of this noncritical social support may have stress-buffering effects [20]. Furthermore, in a stressful environment, interaction with dogs may provide detainees with a source of pleasure, relaxation, and connection to the outside world [20,21], which may also decrease stress.

#### 1.3.2. Self-Esteem

When training the dog, detainees are trusted with responsibility, which can enhance a sense of autonomy and competence [22]. Achieving the training goals with the dogs may increase participants’ self-esteem [23]. Hill [10] pointed out that being able to train and interact with a non-judging animal may help detainees to perceive themselves as someone who can take responsibility and do good for others.

### 1.4. The HAB in a DTP

Although it is often suggested that the HAB decreases stress and increases self-esteem, this has seldomly been examined in DTPs. Menna and colleagues [16] state that the HAB plays a crucial role within Animal-Assisted Interventions. Some aspects of the HAB within DTPs may be of particular importance in the presumed association with stress and self-esteem. Detained juveniles have often experienced rejection, lack of social support, and other social adversities [22]. In DTPs, detainees can interact with someone with no interest in their past mistakes [9] and can experience unconditional acceptance and companionship [24]. Therefore, perceived reciprocity (feeling liked by the dog) may be of particular importance for detained juveniles.

### 1.5. Current Study 

The goal of the current study was to examine the presumed role of the HAB and to answer the following question: To what extent does the HAB have a positive impact on stress and self-esteem of juveniles participating in the Dutch Cell Dogs training?

Both the quality of the HAB and the perception of reciprocity were included. We expected to find that a stronger HAB was related to higher self-esteem and lower stress, at the same timepoint and over time. This study expands previous work by studying an often assumed, but seldom investigated mechanism: the HAB in DTPs. By combining quantitative and qualitative data, and by using variable-centered and person-centered approaches, the study aims to improve the understanding of a hypothesized working mechanism of DTPs.

## 2. Materials and Methods

### 2.1. Participants

For this study, data of a quasi-experimental study on the effectiveness of DCD were used [14]. A quasi-experimental design was chosen because of the small number of detainees per facility applying for the DCD program, therefore randomly allocating participants to an experimental or control group would have resulted in smaller training groups than intended within the DCD program [8]. Because the current study specifically focused on detained juveniles participating in DCD, only participants in the intervention group aged 12 to 25 years old were included (*n* = 87). All participants also received treatment as usual (e.g., Multi-systematic Therapy, etc.), DCD was implemented as an additional program.

Participants were recruited in secured residential facilities in the Netherlands, offering treatment for severe behavioral problems. Data were collected between 2016 and 2019, at three timepoints: pre-training (T1), four weeks after the start of the training (T2), and at the end of the training (T3). Due to missing interview data on all timepoints, 12 participants were excluded from the analyses. There were various reasons for the missing interview data; for instance, some participants only wanted to participate in the questionnaires, and not in the interviews, or there was not enough time left for the interview because participants had other obligations.

The average age of remaining participants (*n =* 75) at T1 was 19.5 years (*SD* = 3.12, range = 12.9–25.5). In Table 1, participants’ characteristics are shown. Chi-square tests and an independent samples *t*-test showed no significant differences between the excluded and included participants in terms of background variables (i.e., gender, cultural and educational background, type of facility, and age).

Of the 75 participants, 64 juveniles successfully completed DCD. Five participants dropped out of the program, for six participants it was unknown whether they successfully have completed DCD. Attrition analysis was conducted to investigate whether detainees who participated in all three waves differed from those who did not. Chi-square tests revealed no significant differences in gender, type of index offense, cultural background, or type of facility. A significant difference was found for educational level, χ^2^(3) = 8.00, *p* = 0.046. Detainees who participated in all three waves were more likely to have partaken in secondary education instead of primary or tertiary education, χ^2^(1) = 4.90, *p* = 0.027. Finally, independent samples *t*-tests showed no significant differences in outcome variables on baseline (stress, *p* = 0.640, and self-esteem, *p* = 0.412).

### 2.2. Procedure

Participants of the study were recruited during the first meeting between DCD staff and detainees. Written informed consent was obtained from all participants before participation. Questionnaires and interviews were taken in a private room in the facility where the juvenile was residing. Participants received a small gift (e.g., phone card, candy, or prison store credit) for participating at each timepoint. Study procedures were approved by the Ethical Committee of the Faculty of Social and Behavioral Sciences of the University of Amsterdam (No. 2015-CDE-6363).

#### Dutch Cell Dogs 

DCD has been implemented in various correctional facilities across the Netherlands since 2009. Every detained juvenile meeting the inclusion criteria for DCD was eligible for the study. Inclusion criteria for DCD were the following: detainees could participate when they (a) were physically able to participate in the training and strong enough to walk a dog, (b) were sufficiently mentally alert (despite potential medication use) to follow the instructions of the DCD staff, and (c) remained in the respective facility for at least two months after the start of the training [8].

For the dogs, there were no inclusion criteria in terms of breed or age. All dogs participating in the program were shelter dogs and had behavioral problems because of previous experiences (e.g., neglect). The only inclusion criterion for the shelter dogs was that they had to be interested in treats, given the reward-based training methods [25].

The training group consists of six detainee–dog dyads, which remain the same throughout the training. During the first meeting, the DCD staff observes participants’ behavior while discussing what is expected from participants. Based on these observations, DCD staff matches each participant with their own shelter dog (for example, an energetic person is matched with an active dog [25]). If possible, the DCD staff also matches detainees and dogs based on shared experiences, such as neglect [11].

Detainees train “their” dogs for eight weeks: twice a week for a two-hour session. At the end of each training session, there is some time for relaxation: participants groom or play with the dog before the dogs return to the shelter. The dogs are brought to the correctional facility for the training sessions and return to the shelter after the session. The aims of DCD are (1) to socialize the behaviorally challenging shelter dogs and thereby increase their chance of adoption, (2) to increase self-esteem and social and communicative skills of detainees, and (3) to decrease recidivism [11]. During the training sessions, the dogs’ welfare and protection is assured by two DCD staff (certified instructors) who are continuously present when participants interact with the shelter dogs. A more elaborate description of DCD can be found in the study protocol [8].

### 2.3. Measures

To investigate the hypotheses, both quantitative and qualitative data were used. Measuring the HAB in a DTP is methodologically challenging, given that most existing assessment methods focus on pet owners. By using structured interviews about the detainees’ experiences with DCD, we were able to measure the HAB quality as experienced within the specific DTP context.

#### 2.3.1. HAB

The HAB was assessed at T2 and T3: the HAB quality by structured interviews and perceived reciprocity of the HAB by self-report questionnaire items.

HAB Quality. Responses to interview questions that yielded information about the HAB quality were selected to transcribe and code. Originally, the interview at T2 consisted of 15 questions and the interview at T3 of 19 questions. Seven interview questions of T2 and nine interview questions of T3 were selected, for example: “Did you learn something from the dog about yourself?” The selected questions are presented in Appendix A, Table A1. The complete set of interview questions is available upon request.

The answers were extensively discussed and summarized into categories describing aspects of HAB, based on recent literature on the HAB and DTPs, e.g., [26]: *Positive Emotions* (describing positive emotions or emotional benefits from interaction with the dog, for example, “I like spending time with the dog, it is great”); *Self-Awareness* (describing what he/she learned from the dog, comparing self to the dog and vice-versa, for example, “To stay calm and alert, because a dog can change its behavior anytime”); *Benefits for the Dog* (describing benefits of the training for the dog, for example; when asked to compliment the dog; “You [the dog] did so well, you are now less jumpy than before”); *Interest* (expressing interest into the past, future and/or feelings of the dog when asked which three questions (s)he would like to ask the dog, for example; “What did you experience when you were younger?”); and *Friendship* (describing the kind of bond: “indifferent”, “trainer”, or “friend”).

For each interview, participants were assigned a score on these five categories ranging between 0 and 4, with 4 indicating high quality of the HAB. Furthermore, overall affection was scored from 0 to 9, a higher score indicating higher affection. More detailed information about the coding categories can be found in the coding manual (available upon request).

A subset of 15 interviews was independently coded by the first, second and fifth author to check interrater-reliability. Fleiss’ Kappa was 0.431 (0.429–0.433) for T2 and 0.452 (0.449–0.454) for T3. Overall, Fleiss Kappa was 0.450 (0.449–0.452), which is considered to be moderate [27]. The remaining interviews were coded by the first author.

Perceived Reciprocity. The Pet Bonding Scale [28] (PBS) was used to measure the perceived reciprocity in the HAB. The PBS consists of 25 items and assesses one’s attachment to his/her companion animal. Five items that are applicable to the participants of DCD (i.e., trainer instead of owner) and that reflect perceived reciprocity were selected: “The dog loves me”, “The dog misses me when I am gone”, “The dog loves me no matter what”, “The dog stays close to me when I am upset”, and “The dog has feelings”. The items were rated along a 3-point Likert-type scale *never/no* (1) to *always/yes* (3). Cronbach’s alpha for these items was acceptable, ranging between 0.65 and 0.70.

#### 2.3.2. Outcome Measures

Stress was measured at all three timepoints by using 10 items of the Perceived Stress Scale [29] (PSS). An example of these items is, “In the last month, how often have you felt nervous and stressed?” Items were answered on a 5-point Likert-type scale, ranging from *never* (0) to *very often* (4). Internal consistency for this scale was found to be good across waves, with Cronbach’s alpha’s ranging between 0.80 and 0.82.

Self-esteem was assessed at all three timepoints by using the Rosenberg’s Self Esteem Scale [30] (RSES). The RSES consists of 10 items, for example: “On the whole, I am satisfied with myself” Items were answered on a 4-point Likert-type scale ranging from *totally disagree* (0) to *totally agree* (3). Internal consistency for the total self-esteem scale was found to be good across waves, with Cronbach’s alpha’s ranging between 0.87 and 0.90.

### 2.4. Statistical Analyses

Data were analyzed using Structural Equation Modeling (SEM) in Mplus 8.5 [31]. Maximum information likelihood with standard errors and chi-square robust to non-normality (MLR) was used in all models.

Both variable-centered and person-centered approaches were applied. Variable-centered approach analyses, using average scores to estimate relations between variables, are useful in describing overall associations. However, this approach does not take heterogeneity within the sample into account. To identify individual differences, a person-centered approach is useful, in which individual scores are used to identify different groups representing different patterns of change in stress and self-esteem.

#### 2.4.1. Factor Structure and Measurement Invariance of HAB Measures 

Latent factors of HAB quality and perceived reciprocity at T2 and T3 were created by using Confirmatory Factor Analyses (CFA) in Mplus. A one-factor model on T2 was estimated to test whether the items loaded on a single factor. Second, measurement invariance was tested to confirm that the one-factor model of the HAB was equivalent across the two measurement occasions. Three consecutive and nested CFA models with increasing equality constraints on factor loadings and indicators’ intercepts were run. The most restricted model that still showed adequate model fit was rerun using the effect coding method as proposed by Little and colleagues [32], to create meaningful latent means and variances. By constraining the set of factor loadings to average 1 and the set of indicator intercepts to sum up to 0, latent means and variances that reflect the observed metric of the underlying items are estimated. Factor scores were saved for subsequent analyses.

#### 2.4.2. Descriptives 

Descriptive statistics were obtained in IBM SPSS Statistics 26. Pearson correlations were computed for the included study variables. Baseline differences in stress and self-esteem, and differences in HAB quality and perceived reciprocity were tested for sex, ethnicity and type of facility using independent-samples *t*-tests and chi-square difference tests. Baseline differences for age were tested by computing correlations. To assess overall changes in stress, self-esteem and HAB indicators during the program, paired samples *t*-tests were conducted.

#### 2.4.3. Variable-Centered Analyses 

Cross-Lagged Panel Models (CLPMs) were used to investigate concurrent and over time associations between the HAB and stress and self-esteem. Cross-lagged effects were tested to examine the hypotheses that a stronger HAB is related to lower stress and higher self-esteem over time. The analyses were run separately for HAB quality and perceived reciprocity, and for the outcome variables stress and self-esteem.

#### 2.4.4. Person-Centered Analyses

Growth Mixture Modeling (GMM) was used to examine heterogeneity in trajectories of change in self-esteem and stress across the three timepoints, and whether these different trajectories were predicted by differences in the HAB. First, general change in stress and self-esteem was established using Latent Growth Curve (LGC) modeling. When the model showed adequate fit to the data, GMM was conducted by a three-step approach [33].

In the first step, the model was run without predictors, to determine the number of latent classes within trajectories of stress and self-esteem. Several models with increasing numbers of classes were estimated to decide on the best model solution. In the second step, the most likely class variable was created, representing participants’ probability of belonging to each of the classes. Finally, the most likely class was regressed on the predictor variables: HAB quality and perceived reciprocity at T2 and T3.

## 3. Results

### 3.1. Preliminary Analyses

#### 3.1.1. Factor Structure and Measurement Invariance of HAB Measures

The one-factor model with the six interview coding categories on T2 showed reasonable fit to the data, χ^2^/*df* ratio = 1.69, Comparative Fit Index [CFI] = 0.97, Root Mean Square Error of Approximation [RMSEA] = 0.10. Standardized indicators’ loadings onto the factor were all statistically significant and ranged from 0.44 to 0.91. The model with configural invariance (Model 1) showed good model fit. Constraining the factor loadings (Model 2) and the intercepts (Model 3) to be equal across time did not significantly worsen model fit.

The one-factor model with the five PBS items on T2 showed good fit to the data, χ^2^/*df* ratio = 0.62, CFI = 1.00, RMSEA = 0.00. Standardized indicator loadings onto the factor were all statistically significant and ranged from 0.37 to 0.90. The model with configural invariance (Model 1a) did not show adequate model fit. As the modification indices suggested, a correlation between question 7 (“The dog misses me when I am gone) at T2 and T3 was added. The adjusted model (Model 1b) showed good fit to the data. Constraining the factor loadings (Model 2) and the intercepts (Model 3) to be equal across time did not significantly worsen model fit. Model fit indices for all described models testing measurement invariance are presented in Appendix B, Table A2 and Table A3.

For both the interview data and PBS items, Model 3 was chosen and rerun using the effect coding method. The resulting factor scores, representing HAB quality and perceived reciprocity, were saved for further analyses.

#### 3.1.2. Descriptives

The means, standard deviations, skewness, kurtosis, and correlations between all variables are shown in Table 2. On all timepoints, more stress was correlated with lower self-esteem. Perceived reciprocity and HAB quality were positively correlated, except for perceived reciprocity at T2 and HAB quality at T3. No significant correlations between the HAB indicators and stress or self-esteem were found. Skewness and kurtosis for all variables were within acceptable range, except for kurtosis for stress at T3, which shows moderate non-normality [34].

*T*-tests on baseline scores of stress, self-esteem, and HAB indicators showed no significant differences with regard to ethnicity and sex, except for the baseline level of stress, which was higher for girls (*M* = 2.17, *SD* = 0.57) than for boys (*M* = 1.53, *SD* = 0.80), *t*(73) = 2.44, *p* = 0.017. Chi-square tests showed no significant differences on baseline variables with regard to type of facility, and no significant correlations with age were found. Sex was added as a control variable for stress.

The *t*-tests revealed no significant differences between T1 and T3 for stress or self-esteem, and no significant differences between T2 and T3 for HAB quality. Perceived reciprocity was significantly higher at the end of the training compared to halfway the training, *t*(67) = 3.34, *p* = 0.001.

### 3.2. Variable-Centered Analyses 

#### 3.2.1. Stress

**Figure 1:**
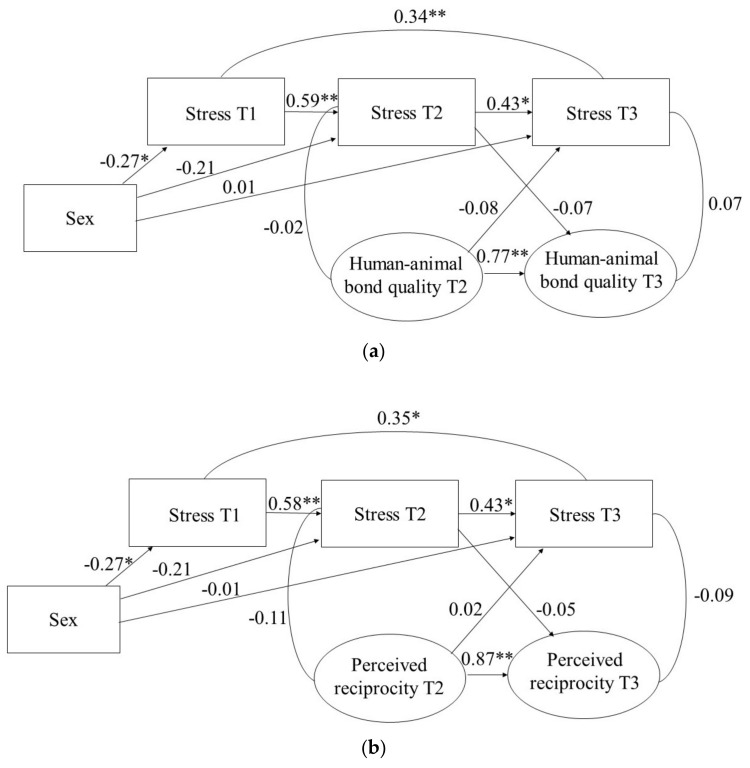
(**a**) CLPM for Stress and Human–Animal Bond Quality, and Sex as a Covariate. (**b**) CLPM for Stress and Perceived Reciprocity, and Sex as a Covariate. Note. * *p* < 0.01 ** *p* < 0.001.

To examine the effect of the HAB on stress, CLPMs with sex as a covariate were estimated. The model demonstrated good fit to the data for HAB quality (χ^2^/*df* ratio = 0.82, CFI = 1.00, RMSEA = 0.00) and for perceived reciprocity (χ^2^/*df* ratio = 0.66, CFI = 1.00, RMSEA = 0.00). For both models, a stability path from stress at T1 to stress at T3 had to be added for adequate model fit. The model results are shown in Figure 1a,b. In both models, the autoregressive paths were significant, indicating that stress, HAB quality, and perceived reciprocity were stable over time. Sex was significantly related to stress at T1 (β = −0.27, *p* = 0.003) in both models, but not to stress at T2 and T3. Finally, none of the cross-sectional or prospective (cross-lagged) associations between the HAB and stress were significant.

#### 3.2.2. Self-Esteem

**Figure 2:**
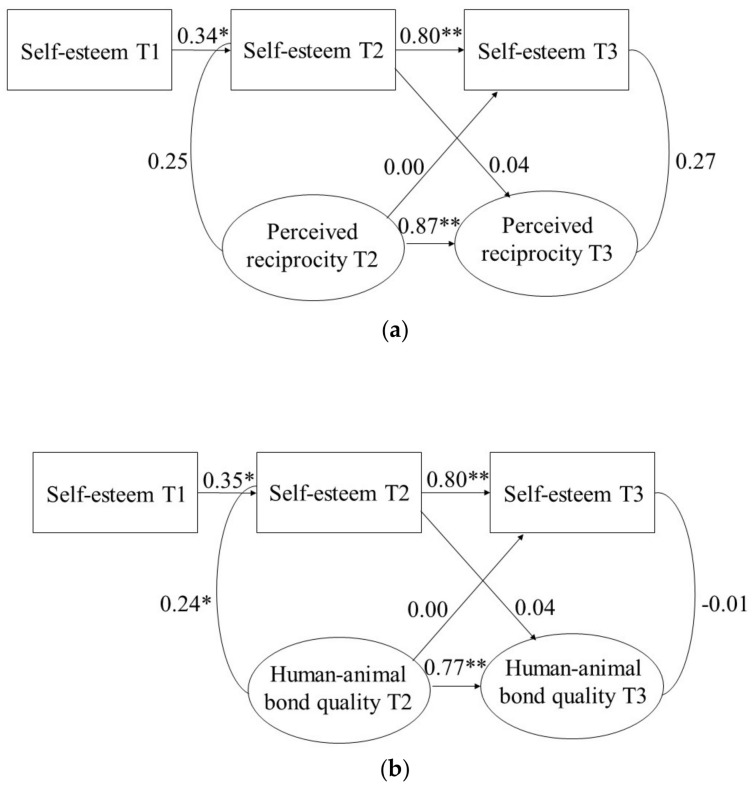
(**a**) CLPM for Self-Esteem and Human–Animal Bond Quality. (**b**) CLPM for Self-Esteem and Perceived Reciprocity. Note. * *p* < 0.05. ** *p* < 0.01.

To examine effects of the HAB on self-esteem, CLPMs were estimated. The CLPM demonstrated good fit to the data for HAB quality (χ^2^/*df* ratio = 0.59, CFI = 1.00, RMSEA = 0.00) and perceived reciprocity (χ^2^/*df* ratio = 1.28, CFI = 0.99, RMSEA = 0.06). The CLPMs are shown in Figure 2a,b. In both models, the autoregressive path coefficients were significant, indicating that self-esteem, HAB quality, and perceived reciprocity were stable over time. Of the cross-sectional associations, only for HAB quality and self-esteem at T2 a significant positive association was found (β = 0.24, *p* = 0.041). None of the cross-lagged effects were significant.

### 3.3. Person-Centered Analyses

#### 3.3.1. Stress

**Latent Growth Curve.** A LGC model for stress was estimated to analyze general development over time. The model for stress showed good fit to the data, χ^2^/*df* ratio = 0.80, CFI = 1.00, RMSEA = 0.00. The estimated mean of the intercept, indicating the initial level of stress, was 1.59 (*SE* = 0.09, *p* < 0.001). Significant variance of the intercept (σ = 0.41, *SE* = 0.10, *p* < 0.001) indicated individual differences in the initial level of stress. The estimated mean of the slope was nonsignificant (*p* = 0.118), indicating that on average, there was no change in stress over time. Nonsignificant variance of the slope (*p* = 0.922) indicated that there were no individual differences in change in stress over time. Therefore, no latent class analysis was conducted for stress.

#### 3.3.2. Self-Esteem

**Latent Growth Curve.** The LGC model for self-esteem showed good fit to the data, χ^2^/*df* ratio = 0.69, CFI = 1.00, RMSEA = 0.00. The residual variance of self-esteem at T3 was fixed to zero because of a small, nonsignificant negative residual variance. The estimated mean of the intercept, indicating the initial level of self-esteem, was 2.19 (*SE* = 0.06, *p* < 0.001). Significant variance of the intercept (σ = 0.10, *SE* = 0.07, *p* = 0.014) indicated individual differences in the initial level of self-esteem. The estimated mean of the latent linear slope, indicating significant decrease in self-esteem across the three timepoints, was −0.08 (*SE* = 0.04, *p* = 0.042). Furthermore, significant variance of the latent slope was found, indicating individual difference in change in self-esteem across the three timepoints (σ = 0.07, *SE* = 0.03, *p* = 0.006).

**Growth Mixture Modeling.** GMM was used to classify the detained juveniles into latent groups, based on their initial level of self-esteem and the development over time. Model fit indices are shown in Table 3. Based on these indices, the two-class solution was chosen.

**Figure 3:**
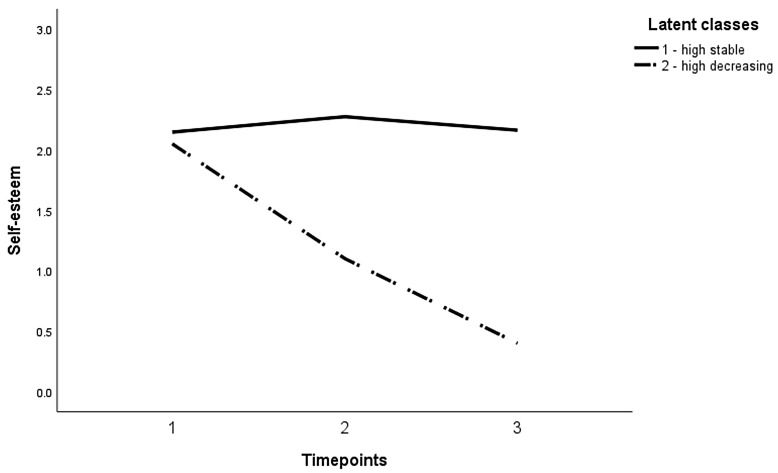
Graphical Representation of the Identified Classes.

The two classes represented two different patterns of change in self-esteem. The majority of detained juveniles (90.54%, *n* = 67) had a relatively high mean score for self-esteem: 2.20 (*SE* = 0.06, *p* < 0.001) and no change over time, as indicated by a nonsignificant latent slope mean (*p* = 0.813). A minority of the detained juveniles (9.46%, *n* = 7) had a relatively high mean score for self-esteem (*M* = 2.11, *SE* = 0.15, *p* < 0.001), which significantly decreased over time (*b* = −0.86, *SE* = 0.10, *p* < 0.001). The first class was labelled as “high stable” and the second class as “high decreasing”. The different patterns are visualized in Figure 3. The regression of the most likely class variable on the HAB predictors showed that the HAB quality was not a significant predictor of class membership at T2 (*p* = 0.371) or at T3 (*p* = 0.862), nor perceived reciprocity at T2 (*p* = 0.275) or at T3 (*p* = 0.135).

## 4. Discussion

The present study was one of the first addressing the role of PBDP’s underlying mechanisms, by aiming to answer the question: To what extent does the HAB have a positive impact on stress and self-esteem among detained juveniles participating in DCD?

To our knowledge, no HAB assessment instruments specific to the DTP context exist. Given the aims and the program characteristics of DCD, some often used indicators in studies including pet owners do not apply to the DTP context (e.g., expending efforts and resources). Other aspects that may be unique for the HAB within a DTP (e.g., training benefits for the dog) may not be captured by existing instruments. Therefore, we used interview and questionnaire data to assess the HAB within DCD. Six aspects of the HAB quality within DTPs were identified: Positive Emotions, Self-Awareness, Benefits for the Dog, Interest, Friendship, and Overall Affection. Furthermore, perceived reciprocity—assessed as the experience of feeling loved by the dog—was expected to be particularly beneficial for detained juveniles, e.g., [9]. Small to moderate correlations showed that higher HAB quality was related to higher perceived reciprocity on all timepoints, except for HAB quality at T3 and perceived reciprocity at T2.

Contrary to our expectations, HAB indicators did not predict stress or self-esteem over time. Only for the HAB quality and self-esteem at T2, a significant concurrent association was found, suggesting that higher HAB quality was related to higher self-esteem one month after the start of DCD. Furthermore, differences in patterns of self-esteem (“high stable” and “high decreasing”) could not be explained by HAB differences. On average, the level of stress did not change during DCD, and no individual differences in change in stress over time were found.

Overall, these findings were somewhat surprising, given that the HAB has frequently been suggested as a mechanism of change in PBDPs, e.g., [22]. However, this study is one of the first to empirically investigate the association of the HAB and stress and self-esteem within a DTP. Suggestions that the HAB is a working mechanism in PBDPs are often based on studies including long-lasting Animal-Assisted Interventions (AAIs), in which the animal is more integrated in the participants’ life, or studies including pet-owners, e.g., [35]. However, the HAB in DTPs may differ given the specific program characteristics. The DCD training lasted for only eight weeks and detainees had only access to the dog during the training sessions. Moreover, DCD strongly focuses on changing the dogs’ behavior, whereas AAIs mainly focus on behavioral change in the participant. It could be reasoned that in a short period of time, positive human–animal interactions do not translate to improvements in well-being.

Importantly, on average, relatively stable low levels of stress and relatively stable high levels of self-esteem among the detained juveniles were found, making change over time less likely. One explanation of these levels may be that participants also received psychological therapy. The high stability showed that stress and self-esteem did not vary much over time and therefore little variance is left to be explained. Together with the findings of Duindam and colleagues [14]—no differences between DCD and the control group in change over time for stress and self-esteem—these findings suggest that on average, DCD, and more specifically the HAB, does not have an effect on stress and self-esteem of detained juveniles.

However, it should be noted that stress and self-esteem were reported about a longer period of time (e.g., a month). Participants may have experienced beneficial psychosocial effects during the training sessions. The interaction with the dog during the training sessions may have a short-term stress-buffering effect by providing a break from a stressful environment [20] and a source of pleasure [21]. In interviews, juveniles mentioned that participating in DCD offered distraction. For example, one participant answered to the question, “What do you like?”: *“Everything, spending time with the dog, it is just a great use of time. In the same position as the dog: it is better than being in your cell”*. However, such beneficial effects might not last when the training is over and the dog returns to the shelter. Therefore, these short-term effects may not be reflected in questionnaires considering a longer period of time.

### 4.1. Limitations

Some limitations of the current study should be considered. First, only a relatively small number of participants was included (*n* = 75), since a very specific sample was targeted: Dutch detained juveniles younger than 26 years old, participating in DCD. The sample size may have had implications for the findings, as a small sample size decreases the statistical power to detect effects. Therefore, the findings should be interpreted with caution. However, the current study was conducted in clinical practice under representative conditions. Given that Weisz and colleagues [36] indicated that a sample size of *n* = 22 was common in psychotherapy research with real clients, the sample size is satisfactory.

Second, given the unique targeted sample, the present findings may not be generalizable to other PBDPs. Differences in duration of the program or in the dog access may result in differences in the HAB and its effect on psychosocial outcomes.

Finally, assessment of the HAB within DTPs is still at an early stage, given that measurement methods of the HAB mainly focus on pet owners. There may be other important aspects of the HAB within a DTP that were not assessed by the interviews and questionnaire items. For example, previous studies have shown that physical contact (e.g., hugging) with a dog may reduce stress, e.g., [37]. Furthermore, there may be differences between dogs’ social behavior towards humans, especially since the dogs included in DCD were behaviorally challenging shelter dogs.

### 4.2. Future Directions

Empirical evidence of the often-presumed role of HAB on psychosocial outcomes in PBDPs is still limited. Exploring other HAB aspects, such as physical contact and the influence of the dog’s behavior, will increase understanding of the role of the HAB within DTPs. Furthermore, including characteristics of the dog—such as its temperament and age—may gain more insight into the HAB. In line with these recommendations, an important direction for future research is the development of a questionnaire assessing the HAB within DTPs. We developed our own measurement method because there was no existing questionnaire applicable to DTPs or PBDPs available. Optimizing measurement methods would be a valuable addition to this field, since it will allow more research with larger samples that include other PBDPs, which is needed to determine the effects of the HAB in PBDPs on psychosocial well-being more accurately.

An interesting area for future research is to examine other psychosocial outcomes in relation to the HAB in DTPs, such as self-control and perceived quality of life. Although no significant effect was found on stress and self-esteem, it would be preliminary to conclude that participants did not benefit from interacting with the dog. In interviews, many juveniles mentioned they enjoyed participating in DCD and experienced benefits of the HAB, in line with previous qualitative data, e.g., [21]. To the question, “What do you like?”, many participants explicitly mentioned (interaction with) the dog. For example, one participant said: *“The relationship you build with the dog, the difference that you see in the dog and yourself (I became much more patient and have almost no fear for dogs anymore), and that you really get to look forward to it*”.

Furthermore, the association between the HAB and self-control is an interesting area for future research, as many juveniles mentioned learning patience from interacting with the dog and self-control is an often-reported psychosocial outcome of DTPs, e.g., [16]. Furthermore, future research on the HAB in DTPs should examine short-term psychosocial outcomes, for example, by using an experience sampling method during the training sessions. It could be reasoned that by providing short-term distraction and relaxation, the HAB has a positive effect on juveniles’ perceived quality of life.

In general, more research on the working mechanisms of DTPs is needed to increase understanding of how DTPs work and to answer the question proposed by Marino [38]: How important is the animal, instead of another novel stimulating component, in DTPs?

## 5. Conclusions

In conclusion, this study demonstrated that the HAB in DCD did not predict detained juveniles’ stress or self-esteem. It was one of the first studies to investigate the role of the HAB within a DTP. By using both qualitative and quantitative data, and by combining a person-centered and variable-centered approach, the current study provides valuable information concerning the role of the HAB within a DTP. More research is needed to determine possible psychosocial benefits. Therefore, we hope that this study stimulates future research on the HAB in DTPs to assess its broader effects.

## Figures and Tables

**Table 1:** Descriptive Statistics for the Final Sample (*n* = 75).

Participants’ Characteristics	%	*n*
Gender		
Male	86.7	65
Female	13.3	10
Type of index offense		
(Attempted) homicide	9.3	7
Violent behavior	29.3	22
Theft or fraud	2.7	2
Sexual offence	10.7	8
Other and unknown	32.0	24
Residential youth care	16.0	12
Cultural background		
Native Dutch	42.7	32
1st or 2nd generation immigrant	57.3	43
Educational background		
Primary education	13.3	10
Secondary education	38.7	29
Tertiary education	28.0	21
Other or unknown	20.0	15
Type of facility		
Youth correctional ^a^	64.0	48
Secure residential youth care ^b^	16.0	12
Adult correctional	20.0	15
Psychiatric diagnosis		
Yes	49.3	37
No	13.3	10
Unknown	37.3	28

^a^ = placement is enforced by juvenile penal law for 12- to 23-year olds; ^b^ = placement is enforced by civil law for 12- to 18-year olds.

**Table 2:** Descriptive Statistics and Correlations.

Variables	1	2	3	4	5	6	7	8	9	10
1. Human–animal bond quality T2	-									
2. Human–animalbond quality T3	0.77 *	-								
3. Perceived reciprocity T2	0.44 **	0.23	-							
4. Perceived reciprocity T3	0.44 **	0.28 *	0.87 **	-						
5. Stress T1	−0.03	0.03	−0.05	−0.08	-					
6. Stress T2	−0.02	−0.07	−0.10	−0.13	0.63 **	-				
7. Stress T3	−0.12	−0.08	−0.04	−0.09	0.62 **	0.61 **	-			
8. Self-esteem T1	−0.06	−0.02	0.05	0.03	−0.51 **	−0.38 **	−0.27 *	-		
9. Self-esteem T2	0.18	0.15	0.15	0.14	−0.24 *	−0.35 **	−0.34 **	0.34 **	-	
10. Self-esteem T3	0.15	0.12	0.07	0.17	−0.48 **	−0.40 **	−0.49 **	0.42 **	0.74 **	-
Mean (*SD*)	2.66 (0.84)	2.67 (0.70)	1.53 (0.34)	1.59 (0.28)	1.62 (0.80)	1.48 (0.73)	1.50 (0.73)	2.18 (0.56)	2.15 (0.66)	2.00 (0.68)
Skewness	−0.23	0.19	−0.64	−0.87	0.79	0.31	0.83	−1.04	−1.14	−0.99
Kurtosis	0.04	−0.45	−0.82	−0.18	0.65	−0.21	2.21	1.94	1.66	1.14

*Note.* * *p* < 0.05. ** *p* < 0.01.

**Table 3:** Model Fit Indices for All Growth Mixture Models for Self-Esteem.

Solution	SSA BIC	Entropy	Adjusted LMR-LRT	Class Size (*N*)		
				1	2	3
One-class	333.35	-	-	74 ^a^		
**Two-class**	**313.52**	**0.94**	**<0.001**	**68**	**6**	
Three-class	305.04	0.94	0.08	63	5	6

Note. Bold part is the chosen model. SSA BIC = sample-size adjusted Bayesian information criterion; LMR-LRT = Lo–Mendell–Rubin likelihood ratio test. Class size represents number of participants within every class, based on their most likely class membership. ^a^ One participant was excluded from the analyses, due to missing values for self-esteem on all timepoints.

## Data Availability

Not applicable. Given the sensitive data, we are not able to share the dataset.

## Appendix A

**Table A1:** Selected Interview Questions reflecting Human–Animal Bond Quality.

Interview Question	Assessment
What do you like [of the training program]?	T2, T3
Picture moment. Imagine you could choose one moment during the training when a picture of you would be taken. Which moment do you choose?	T2, T3
Tell something about the dog you’re training.	T2, T3
What name did you give the dog?	T2
For what reason did you choose this name?	T2
Imagine the dog you’re training could talk. Which three questions would you ask the dog?	T2, T3
Did you learn something from the dog? If yes, what did you learn?	T2, T3
Did you learn something from the dog about yourself?	T2, T3
You’ll have to say goodbye to the dog soon. How do you feel about it?	T3
How was the demonstration?	T3
If you could give a compliment to the dog, which compliment would you give?	T3

## Appendix B

**Table A2:** Model Fit Indices for Testing Measurement Invariance Interview Data.

	χ^2^ (*df*)	χ^2^*/df*	CFI	TLI	RMSEA	AIC	BIC	Scaling Correction Factor MLR	ΔSBχ^2^
Model 1	72.83 (53)	1.37	0.95	0.93	0.07	2221.54	2307.29	0.973	-
Model 2	82.25 (58)	1.42	0.93	0.92	0.08	2221.48	2295.64	0.982	2 versus 1 (5): 9.19, *p* = 0.102
Model 3	87.35 (63)	1.35	0.93	0.93	0.07	2216.03	2278.60	0.977	3 versus 2 (5): 4.96, *p* = 0.420

Note: CFI = comparative fit index; TLI = Tucker–Lewis index, RMSEA = root mean square error of approximation, BIC = Bayesian information criterion, AIC = Akaike information criterion.

**Table A3:** Model Fit Indices for Testing Measurement Invariance PBS items.

	χ^2^ (*df*)	χ^2^*/df*	CFI	TLI	RMSEA	AIC	BIC	Scaling Correction Factor MLR	ΔSBχ^2^
Model 1a	60.44 (34)	1.78	0.80	0.73	0.11	987.36	1056.16	1.040	-
Model 1b	37.21 (33)	1.13	0.97	0.96	0.04	964.97	1036.00	1.034	1b versus 1a (1): 19.75, *p* < 0.01 2 versus 1 (4): 1.23, *p* = 0.873 3 versus 2 (4): 7.00, *p* = 0.136.
Model 2	37.02 (37)	1.00	1.00	1.00	0.00	958.91	1021.05	1.092	
Model 3	43.21 (41)	1.05	0.98	0.98	0.03	956.33	1009.59	1.061	

Note: CFI = comparative fit index; TLI = Tucker–Lewis index, RMSEA = root mean square error of approximation, BIC = Bayesian information criterion, AIC = Akaike information criterion.
